# Dynamic SUMO modification regulates mitotic chromosome assembly and cell cycle progression in *Caenorhabditis elegans*

**DOI:** 10.1038/ncomms6485

**Published:** 2014-12-05

**Authors:** Federico Pelisch, Remi Sonneville, Ehsan Pourkarimi, Ana Agostinho, J. Julian Blow, Anton Gartner, Ronald T. Hay

**Affiliations:** 1 Centre for Gene Regulation and Expression, College of Life Sciences, University of Dundee, Dundee DD1 5EH, UK

## Abstract

The small ubiquitin-like modifier (SUMO), initially characterized as a suppressor of a mutation in the gene encoding the centromeric protein MIF2, is involved in many aspects of cell cycle regulation. The dynamics of conjugation and deconjugation and the role of SUMO during the cell cycle remain unexplored. Here we used *Caenorhabditis elegans* to establish the contribution of SUMO to a timely and accurate cell division. Chromatin-associated SUMO conjugates increase during metaphase but decrease rapidly during anaphase. Accumulation of SUMO conjugates on the metaphase plate and proper chromosome alignment depend on the SUMO E2 conjugating enzyme UBC-9 and SUMO E3 ligase PIAS^GEI-17^. Deconjugation is achieved by the SUMO protease ULP-4 and is crucial for correct progression through the cell cycle. Moreover, ULP-4 is necessary for Aurora B^AIR-2^ extraction from chromatin and relocation to the spindle mid-zone. Our results show that dynamic SUMO conjugation plays a role in cell cycle progression.

## Abstract (web summary)

The ubiquitin-like modifier SUMO is involved in many aspects of the cell cycle, but its dynamics during mitosis are unknown. Here, Pelisch *et al.* use C. elegans to show that SUMO accumulates on the metaphase plate and is required for proper chromosome alignment, and deconjugation is required to progress through the cell cycle.

## Graphical abstract

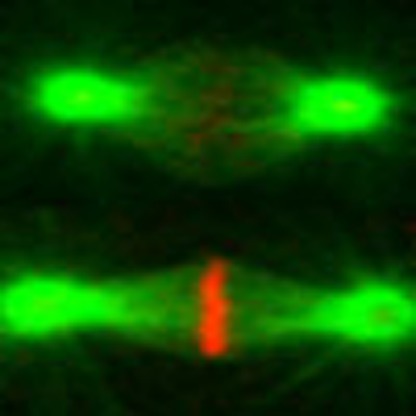

Small ubiquitin-related modifier (SUMO) conjugation is essential for development in mammals^1^,^2^ and in the nematode *Caenorhabditis elegans (C. elegans)*^3^,^4^,^5^,^6^. Mammals contain three different SUMO proteins^7^, whereas, in *C. elegans*, there is one SUMO orthologue, SMO-1 (hereafter, SUMO). Sumoylation occurs through the action of an E1-activating enzyme (the Sae1/Sae2 heterodimer in humans, AOS-1/UBA-2 in worms), an E2-conjugating enzyme (Ubc9 in humans, UBC-9 in worms) and SUMO-specific E3 ligases^7^,^8^. The most studied type of SUMO E3 ligase is the SP-RING E3 ligase family, which includes PIAS proteins in vertebrates and their yeast homologues Siz1, Siz2 (refs 9, 10). Regulation is also achieved at the level of desumoylation by SUMO-specific isopeptidases: SENP1, 2, 3, 5, 6 and 7 in vertebrates^11^,^12^. Post-translational protein modifications including phosphorylation, ubiquitylation and sumoylation are essential for mitotic progression. Although phosphorylation has been particularly well studied, ubiquitylation also controls mitotic progression, either by facilitating proteasome-mediated degradation of proteins or by regulating protein extraction from chromatin^13^,^14^,^15^,^16^,^17^. Early data indicated that SUMO is involved in cell cycle progression. SUMO was initially characterized as a suppressor of a mutation in the gene encoding the centromeric protein MIF2 (refs 18, 19), and the SUMO conjugating enzyme *Ubc9* as well as the SUMO protease *Ulp1* regulates cell cycle progression in yeast^20^,^21^. Later on, several studies showed essential roles for sumoylation in controlling chromosome condensation and cohesion, kinetochore assembly and function, and spindle dynamics^22^,^23^,^24^,^25^,^26^,^27^,^28^,^29^,^30^,^31^,^32^,^33^.

The *C. elegans* embryo is a powerful model system for studying metazoan cell division and it has provided important mechanistic insights into cell cycle progression, particularly related to kinetochore function^34^. SUMO has been shown to play many roles in *C. elegans* including gonadal and vulval development^35^,^36^, translesion synthesis DNA polymerase POLH-1 stability^37^, cytoplasmic intermediate filament assembly^38^ and Hox gene expression^39^. In *C. elegans* the SUMO and Ubc9 orthologues are *smo-1* and *ubc-9,* while the PIAS and *mms21* (ref. 40) orthologues are GEI-17 (ref. 41) and ZK1248.11.1 (hereafter MMS-21), respectively. Four SUMO proteases (ubiquitin-like proteases, ULPs) ULP-1, ULP-2, ULP-4 and ULP-5 have been recognized in *C. elegans*, while ULP-3 is the putative Nedd8 protease (NEDP1) orthologue. As the detailed dynamics of conjugation and deconjugation and the mechanisms of action of sumoylation during mitosis remain unexplored, we took advantage of *C. elegans* to establish the contribution of SUMO to a timely and accurate cell division. We report here that SUMO conjugation increases during metaphase but decreases rapidly during anaphase. The accumulation of SUMO on the metaphase plate and proper chromosome alignment depend on the SUMO E2 conjugating enzyme UBC-9 and SUMO E3 ligase GEI-17. Deconjugation is achieved by the SUMO protease ULP-4 and is crucial for correct progression through the cell cycle. Our results show that highly regulated and dynamic SUMO conjugation plays a major role in cell cycle progression.

## Results

### The sumoylation pathway affects chromosome dynamics

**Figure 1:**
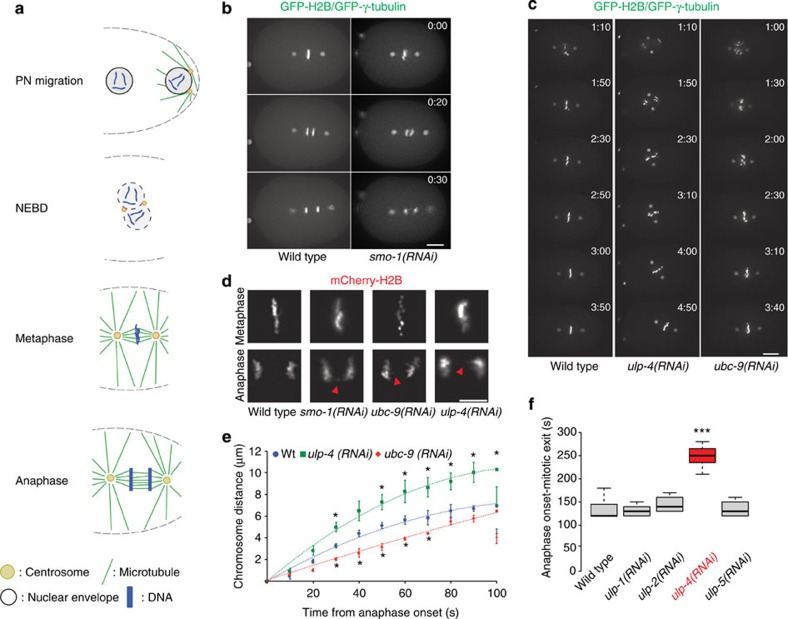
The SUMO conjugation pathway is essential for mitotic chromosome segregation. (**a**) Schematic of the different stages during the first *C. elegans* embryonic cell cycle. (**b**) Knockdown of *smo-1* leads to chromosome alignment and segregation defects. Embryos expressing GFP-H2B and GFP-γ-tubulin were analysed by live imaging. Insets show a × 4 magnification of the DNA mass in both wild type and *smo-1(RNAi)* embryos. Scale bar, 10 μm. The times indicated in the top right of each image of the stack are relative to anaphase onset (min:s). (**c**) Knockdown of *ubc-9 and ulp-4* also leads to chromosome alignment and segregation defects. Embryos expressing GFP-H2B and GFP-γ-tubulin were analysed by live imaging. Scale bar, 10 μm. The times indicated in the top right of each image of the stack are relative to NEBD (min:s). (**d**) Knockdown of *smo-1*, *ubc-9* and *ulp-4* leads to chromosome alignment defect and to the presence of anaphase bridges. Embryos expressing mCherry-H2B were analysed by live imaging. Red arrowheads indicate the chromosome bridges. Scale bar, 5 μm. (**e**) Chromosome segregation was analysed from wild type, *ubc-9(RNAi)* and *ulp-4(RNAi)* embryos. Data are represented as mean and s.d. for each time point (*n*=7 for each condition). (**f**) The time between anaphase onset and mitotic exit was measured and results are shown as a boxplot. Centre lines show the medians; box limits indicate the 25th and 75th percentiles; whiskers extend 1.5 times the interquartile range from the 25th and 75th percentiles. ***P<0.0001 (ANOVA).

The primary advantage of *C. elegans* embryo is that the architecture of the syncytial gonad makes it possible to use RNAi to generate oocytes whose cytoplasm is reproducibly depleted of a defined target protein. The depleted oocytes can then be analysed as they attempt their first mitotic division following fertilization. We took advantage of this feature and used the *C. elegans* first embryonic division to study the role of sumoylation in cell cycle progression. Figure 1a provides a timeline for the first embryonic mitosis and highlights some of its key features. We analysed the first mitotic division using embryos expressing GFP-H2B and GFP-γ-tubulin, allowing visualization of chromatin and centrosomes. Depletion of *smo-1* leads to chromosome mis-alignment and anaphase bridges (Fig. 1b and Supplementary Movie 1). We next depleted *ubc-9* and the SUMO proteases *ulp-1*, *2*, *4* and *5* in embryos expressing GFP-H2B and GFP-γ-tubulin. Depletion of *ubc-9* blocked SUMO conjugation (Supplementary Fig. 1a), while depletion of all the SUMO proteases, but *ulp-5*, increased the presence of SUMO conjugates (Supplementary Fig. 1b). Depletion of *ubc-9* led to chromosome mis-alignment at metaphase (Fig. 1c, right panel). Knockdown of the SUMO protease *ulp-4* also led to chromosome misalignment (Fig. 1c, middle panel). After the pronuclei meet, the nuclear–centrosome complex moves to the centre of the embryo and rotates to align with the long anterior-to-posterior (A-P) axis of the embryo^42^. In the case of *ulp-4(RNAi)* there was a noticeable rotation defect, spindle rotation was delayed and complete alignment of the spindle with the A-P axis was not observed until anaphase onset (Fig. 1c). In addition, the amplitude of spindle oscillations was increased (Supplementary Fig. 2a). *Ubc-9* knockdown also lead to the metaphase being inappropriately oriented, but the phenotype was less severe with lower penetrance (*n*=4/10, data not shown) and the amplitude of spindle oscillations was diminished (Supplementary Fig. 2a). Spindle pole separation in both *ubc-9(RNAi)* and *ulp-4(RNAi)* embryos was diminished compared with wild-type embryos (Supplementary Fig. 2b). By late anaphase/telophase, the length of *ulp-4(RNAi)* embryos was significantly longer than both wild type and *ubc-9(RNAi)* (Supplementary Fig. 2b). A more detailed image of chromosome structure in metaphase and anaphase for the different RNAi utilized is provided in Fig. 1d, using embryos expressing mCherry-H2B. In addition to the alignment defect, we noticed that the degree of chromatin condensation was altered (Fig. 1d). Depleting *ulp-4* caused chromosomes to segregate twofold faster and led to an increase in the distance between chromosomes between 30 and 100 s after anaphase onset (Fig. 1e). *Ubc-9* depletion slowed chromosome segregation by 1.7-fold and led to a decrease in the distance between chromosomes between 30 and 70 s after anaphase onset (Fig. 1e). *Ulp-4* knockdown was accompanied by a delay in mitotic exit, as determined by the time from anaphase onset to chromatin decondensation (Fig. 1f). These results provide evidence that the sumoylation pathway regulates chromosome dynamics in *C. elegans*.

We then analysed whether kinetochore protein recruitment was affected by the knockdown of the SUMO conjugation/deconjugation pathway. In contrast to localized centromeres of vertebrates, *C. elegans* chromosomes are holocentric with kinetochores forming along their entire length^43^. Nonetheless, the structure and composition of *C. elegans* kinetochores is similar to that of metazoans^43^ (Supplementary Fig. 3a). We analysed the recruitment of an upstream protein in the kinetochore assembly cascade, kinetochore-null (KNL)-2 (ref. 44). GFP-KNL-2 association with chromatin was unaffected by the knockdown of *smo-1*, *ubc-9* or *ulp-4* as analysed by time-lapse microscopy (Supplementary Fig. 3b). This is consistent with the fact that knockdown of the components of the SUMO pathway does not lead to KNL phenotype. We then turned our attention to two downstream kinetochore proteins: MIS-12 and HCP-1^CENP-F^. GFP-HCP-1 was still recruited to kinetochore after knocking down *smo-1* and *ubc-9* (Supplementary Fig. 3c), whereas GFP-MIS-12 recruitment was unaffected by knocking down *ubc-9* and *ulp-4* (Supplementary Fig. 3d). These data indicate that no apparent defect with kinetochore protein recruitment takes place on perturbation of the SUMO conjugation pathway. Still, defects in kinetochore proteins other than recruitment and not detected by these assays could be taking place.

### SUMO localization pattern during mitosis

**Figure 2:**
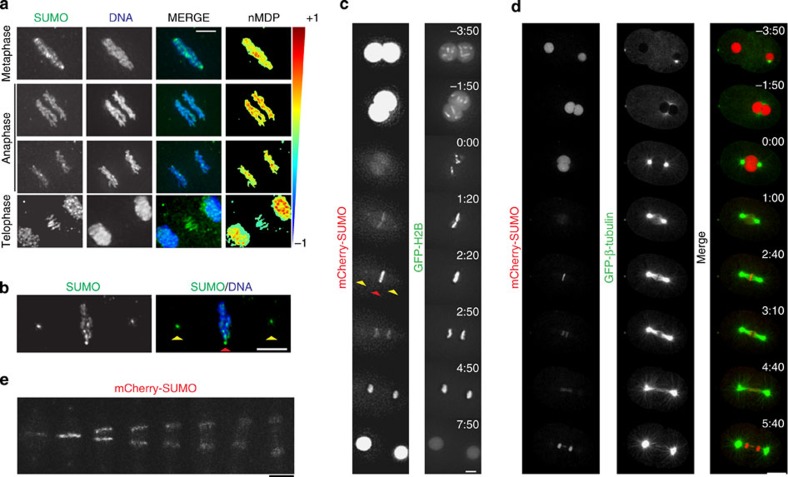
SUMO localization throughout mitosis. (**a**) Embryos at different mitotic stages were analysed by immunostaining using a monoclonal antibody against *C. elegans* SUMO (6F2, See Supplementary Table 2). Quantitation of the co-localization of SUMO with DNA was performed using the normalized mean deviation product (nMDP) values (ranging from −1 to 1). Negative indexes are represented by cold colours (exclusion). Indexes above 0 are represented by hot colours (co-localization)^68^. Scale bar, 2.5 μm. (**b**) An embryo at metaphase was analysed by immunostaining as in (**a**). The red arrowhead marks the metaphase plate, while the yellow arrowhead indicates the centrosomes. Scale bar, 4 μm. (**c**) Embryos expressing mCherry-SUMO(GG) and GFP-tagged H2B were analysed by live imaging on a DeltaVision microscope to study the dynamic behaviour of SUMO localization. Yellow arrows indicate centrosomes and red arrow marks the metaphase plate. Scale bar, 5 μm. The times indicated in the top right of each image of the stack are relative to NEBD (min:s). (**d**) Same as in (**b**) with embryos expressing mCherry-SUMO(GG) and GFP-tagged β-tubulin. Scale bar, 10 μm. The times indicated in the top right of each image of the stack are relative to NEBD (min:s). (**e**) Higher magnification images of mCherry-SUMO from metaphase to telophase are shown. Scale bar, 5 μm.

Having established the need for both SUMO conjugating and deconjugating enzymes in chromosome dynamics, we sought to study SUMO localization by immunostaining. To achieve this, we developed three specific mouse monoclonals and a sheep polyclonal antibody against the only *C. elegans* SUMO orthologue, SMO-1 (Supplementary Fig. 4). SUMO localized to the metaphase plate and also to the centrosomal region (Fig. 2a,b and Supplementary Fig. 5). SUMO also localized to segregating chromosomes at the beginning of anaphase (Fig. 2a). At late anaphase and telophase, SUMO staining was observed in the spindle midzone and in the two daughter nuclei (Fig. 2a). To study the dynamics of SUMO localization in the *C. elegans* embryo, we generated N-terminally fluorescently labelled processed SMO-1 (‘mCherry-SUMO’) under the control of the *pie-1* promoter driving expression in the germline and embryo^45^. mCherry-SUMO was enriched in nuclei and released to the cytoplasm with nuclear envelope breakdown (Fig. 2c and Supplementary Movie 2). In agreement with the behaviour of the endogenous protein, mCherry-SUMO intensity readily increased on chromatin by metaphase (Fig. 2c, red arrowhead). Centrosome staining was also apparent (Fig. 2c, yellow arrowheads). mCherry-SUMO intensity decreased as anaphase progressed and then increased again in the nuclei of daughter cells (Fig. 2c and Supplementary Movie 2). We then performed the same analysis in embryos expressing mCherry-SUMO and GFP-β-tubulin. As observed previously, mCherry-SUMO intensity increased sharply by metaphase and early anaphase, and decreased as anaphase progressed (Fig. 2d). In addition, mCherry-SUMO was localized to the spindle midzone (Fig. 2d). mCherry–SUMO localization from metaphase to telophase is depicted with greater detail in Fig. 2e. These results highlight the dynamic nature of SUMO localization during the first embryonic cell cycle in *C. elegans*.

### SUMO conjugation is dynamically regulated in mitosis

**Figure 3:**
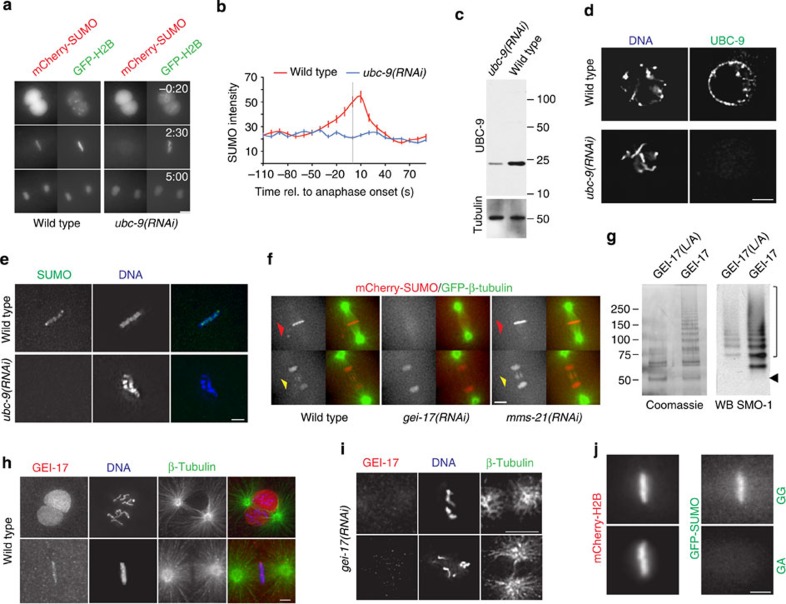
SUMO conjugates concentrate on the metaphasic plate and the spindle midzone and depend on the SUMO E3 GEI-17. (**a**) mCherry-SUMO(GG) and GFP- H2B expressing embryos were treated with control or *ubc-9* RNAi and progression through the first mitotic division was analysed. Scale bar, 5 μm. The times indicated in the top right of each image of the stack are relative to NEBD (min:s). (**b**) Quantitation of SUMO intensity on DNA from the movies depicted in d and e. Data represent the mean intensity (on DNA with background substracted) and s.d. Grey line marks anaphase onset. (**c**) The efficiency of *ubc-9* knockdown was assessed by western blot using an affinity-purified polyclonal antibody. (**d**) UBC-9 localization was assessed with an affinity-purified polyclonal antibody. Samples were labelled with Hoechst 33258. Immunostaining using *ubc-9(RNAi)* embryos is show in the bottom panels. Scale bar, 5 μm. (**e**) Endogenous SUMO labelled with monoclonal antibody (6F2) on the metaphase plate. Scale bar, 2 μm. (**f**) The role of *gei-17* and *mms-21* on SUMO conjugation was addressed in embryos expressing mCherry-SUMO(GG) and GFP-tagged β-tubulin. Images show embryos at metaphase and late anaphase/telophase. The red arrowhead marks the metaphase plate while the yellow arrowhead indicates the spindle midzone. Scale bar, 5 μm. (**g**) *In vitro* auto-sumoylation assay using GEI-17 and the SP-RING mutant GEI-17 ‘L/A’. Black arrowhead, non-modified GEI-17. SUMO-conjugated GEI-17 forms are marked by brackets (top), (**h**) GEI-17 localization was assessed with a rabbit affinity-purified rabbit polyclonal antibody. Samples were labelled with an anti-tubulin antibody and Hoechst 33258. Scale bar, 5 μm. (**i**) Immunostaining was performed as in **h** but using *gei-17(RNAi)* embryos. Scale bar, 4 μm. (**j**) Embryos expressing GFP-SUMO(GG) and the non-conjugatable SUMO(GA), both co-expressing mCherry-H2B were recorded and a representative metaphase is shown for each strain. Scale bar, 2 μm.

To establish whether SUMO staining depends on actual conjugation, we knocked down the SUMO E2 *ubc-9.* This led to the complete loss of the mCherry-SUMO signal at metaphase chromosomes (Fig. 3a,b and Supplementary Movie 3) but not within pronuclei or nuclei (Fig. 3a). UBC-9 protein levels were reduced by ≥80% in embryonic extracts as analysed by western blot (Fig. 3c). Immunostaining with an antibody against UBC-9 localized the protein to the nuclear envelope and on DNA. Specificity of the antibody was confirmed by the lack of fluorescence in *ubc-9(RNAi)* embryos (Fig. 3d). Importantly, knockdown of *ubc-9* also abolished endogenous SUMO staining at metaphase (Fig. 3e).

### SUMO conjugation is mediated by the SUMO E3 ligase GEI-17

Driven by the precisely timed appearance of SUMO conjugates that takes place during mitosis, we turned our attention to putative SUMO E3 ligases. We focused in the PIAS orthologue, GEI-17, and the component of the SMC-5/6 complex, MMS-21. The metaphase- and spindle midzone-specific SUMO conjugation was completely abolished in *gei-17(RNAi)* embryos (Fig. 3f and Supplementary Movie 4). In contrast, *mms-21(RNAi)* embryos behaved like wild-type embryos (Fig. 3f and Supplementary Movie 5). To establish that GEI-17 is a functional orthologue of the Siz/PIAS SUMO E3 ligases, its ability to catalyse the formation of SUMO chains *in vitro* was determined. A fragment of GEI-17 isoform f (aa 133–509) bearing the SP-RING^9^ efficiently forms SUMO chains (Fig. 3g). Mutation of leucine 362 within the SP-RING (equivalent to I363A in yeast^9^) decreased in chain formation (Fig. 3g, ‘GEI-17 L/A’). Immunostaining showed that, like SUMO, GEI-17 is localized to the metaphase plate (Fig. 3h). Importantly, the GEI-17 signal was specific as it was abolished by the *gei-17* RNAi (Fig. 3i). To provide further evidence that the specific SUMO localization is due to the presence of SUMO conjugates and not due to non-covalent association, we showed that the fluorescently labelled non-conjugatable version of SUMO (‘GA’)^46^ fails to accumulate on mitotic chromosomes at metaphase (Fig. 3j). Altogether, SUMO conjugates accumulate on chromatin during metaphase in a manner dependent on not only UBC-9 but also on the E3 ligase GEI-17. However, the accumulation of SUMO is transient, being removed after ~50 s.

### SUMO is deconjugated by the SUMO protease ULP-4^SENP6/7^

**Figure 4:**
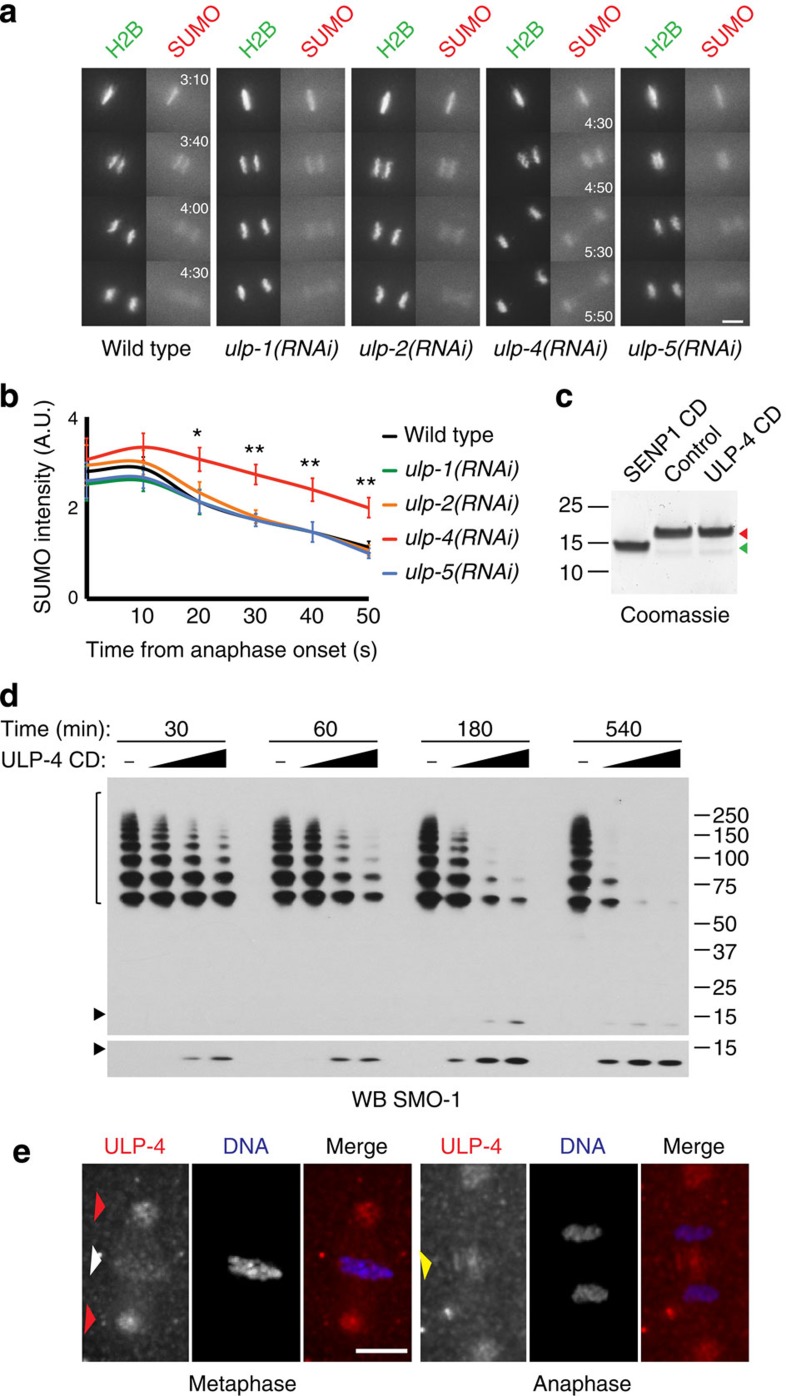
SUMO deconjugation during anaphase is carried out by the SUMO protease ULP-4. (**a**) The effect of knocking down the four putative *C. elegans* SUMO proteases was analysed in mCherry-SUMO(GG) and GFP-tagged H2B expressing embryos. Scale bar, 5 μm. The times indicated in the top right for the wild-type embryo and the bottom right for *ulp-4(RNAi)* embryo are relative to NEBD (min:s). No difference is observed in this timing between wild type and either *ulp-1(RNAi), ulp-2(RNAi)* and *ulp-5(RNAi).* SUMO intensity quantitation is shown in (**b**). Data represent the mean intensity and s.d. of four independent embryos per condition. *P<0.05; **P<0.01 (two-tailed Student’s *t*-test). (**c**) C-terminal hydrolase activity of ULP-4 catalytic domain (‘ULP-4 CD’), as assayed using full-length *C. elegans* SUMO with an HA-tag after the C-terminal diglycine. Green arrowhead, unprocessed SUMO; red arrowhead, processed SUMO. The catalytic fragment of human SENP1 (‘SENP1 CD’) was used as a positive control. (**d**) SUMO chains assembled on GEI-17 are processed by ULP-4. The black arrowhead denotes free SUMO. Brackets mark the SUMO chains assembled on GEI-17. The western blot was developed with a sheep anti-SMO-1 antibody. The lower panel shows a longer exposure to highlight the presence of free SUMO. (**e**) Immunostaining of ULP-4 was performed using an affinity-purified antibody. Images on the left correspond to metaphase, while images on the right, to anaphase. DNA was visualized with Hoechst 33258. Red arrowhead mark centrosomes, white arrowhead marks the metaphase plate, and yellow arrowheads points at the spindle midzone. Scale bar, 5 μm.

Given the phenotypes observed for *ulp-4(RNAi)* in Fig. 1 and the rapid decrease in SUMO conjugation during anaphase, we tested whether SUMO proteases are active at this stage of the cell cycle. To analyse the role of individual SUMO proteases in the accumulation and removal of SUMO from mitotic chromosomes, the expression of each of the four proteases was inhibited by RNAi and the chromatin association of mCherry-SUMO conjugates was followed by time-lapse microscopy. None of the RNAi treatments resulted in a significant increase in SUMO conjugates at metaphase (Fig. 4a,b). Consistent with the data on Fig. 1, SUMO conjugation decreased to a similar extent during anaphase progression in wild type, *ulp-1-*, *ulp-2*- and *ulp-5*-depleted embryos (Fig. 4a,b). In contrast, SUMO was less efficiently removed from its association with chromatin after depletion of *ulp-4* between 20–50 s after anaphase onset (Fig. 4a,b and Supplementary Movies 6 and 7). The ULP-4 catalytic domain is most closely related to those in the mammalian chain-editing enzymes SENP6/7 and, like SENP6/7, ULP-4 was unable to process immature SUMO, although this version of SUMO was efficiently processed by the catalytic domain of human SENP1 (Fig. 4c). ULP-4 actively depolymerizes purified SUMO chains formed by GEI-17 *in vitro* (Fig. 4d). Given its important role during the cell cycle, we sought to determine the localization of ULP-4 by immunostaining. We developed rabbit polyclonal antibodies and showed that ULP-4 localizes around the metaphase plate and the pericentriolar region (Fig. 4e) and in the surroundings of the central spindle (Fig. 4f). In contrast, ULP-1 (orthologue of mammalian SENP1/2) shows no specific enrichment neither at the spindle nor on DNA, but, like SENP1/2 (ref. 25), is enriched in the nuclear envelope (Supplementary Fig. 6).

### SUMO conjugation affects AIR-2 localization

**Figure 5:**
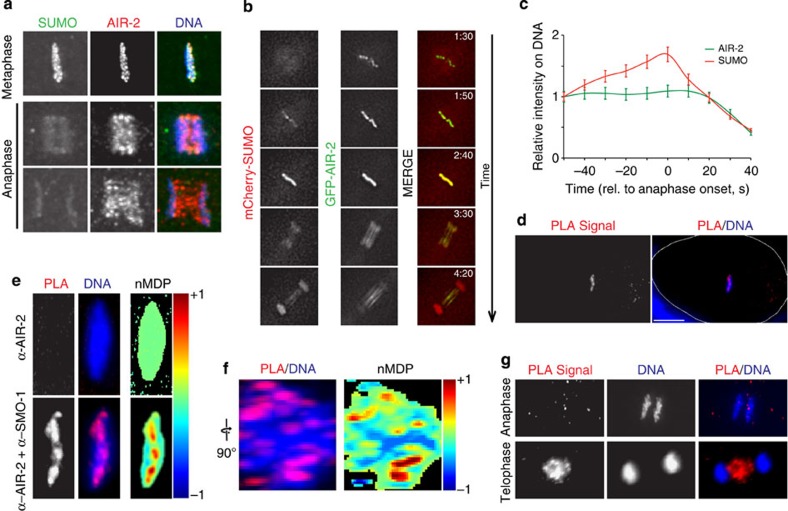
SUMO and AIR-2/Aurora B closely interact in the metaphase plate and spindle midzone. (**a**) Immunostaining of embryos was performed using a monoclonal antibody against SUMO (6F2) and a rabbit polyclonal against AIR-2. DNA was stained with Hoechst 33258. (**b**) The dynamics of SUMO and AIR-2 localization was examined in embryos expressing GFP-tagged AIR-2 and mCherry-SUMO(GG). The times indicated in the top right of each image of the stack are relative to NEBD (min:s). (**c**) Quantitation of the signals in (**b**) is provided in the graph next to the image stack. Data represent mean and s.d. from five independent embryos. (**d**) The image shows an embryo in metaphase subject to the proximity ligation assay (PLA) using a mouse anti-SMO-1 and a rabbit anti-AIR-2 specific antibody. DNA was visualized with Hoechst 33258. (**e**) The same metaphase plate is shown in greater detail, alongside with a negative control in which the anti-SMO-1 antibody was omitted. Quantitation of the co-localization of the PLA signal with DNA was performed using the normalized mean deviation product (nMDP) values (ranging from −1 to 1). Negative indexes are represented by cold colours (exclusion). Indexes above 0 are represented by hot colours (co-localization)^68^ same as in Figure 2. (**f**) The metaphase plate was rotated 90° and the nMDP distribution is shown. (**g**) The SUMO/AIR-2 PLA signal is lost during chromosome segregation and is recovered in late anaphase in the spindle midzone.

We based our search for putative SUMO substrates on the available data for mitotic SUMO substrates or to proteins exhibiting a similar pattern of localization. A strong candidate was the Aurora B orthologue *air-2* (refs 47, 48, 49, 50). Analysis of endogenous SUMO and AIR-2 by immunostaining showed that they co-localize perfectly on aligned metaphase chromosomes and at anaphase onset (Fig. 5a). Translocation of AIR-2 from chromosomes to the spindle midzone was paralleled by the decrease of SUMO staining on DNA (Fig. 5a). To analyse the dynamic behaviour in more detail, embryos expressing GFP-AIR-2 and mCherry-SUMO were analysed. While AIR-2 localized to DNA during prometaphase (Supplementary Movie 8) and metaphase and then translocated to the spindle midzone (Fig. 5b), SUMO co-localized with AIR-2 when chromosomes are aligned on the metaphase plate and in early anaphase (Fig. 5b). Some degree of co-localization was also observed in the spindle mid-zone in late anaphase/telophase (Fig. 5b). AIR-2 translocation to the spindle mid-zone coincided with the loss of SUMO staining during anaphase (Fig. 5b,c and Supplementary Movie 8). To further characterize the SMO-1/AIR-2 interaction, we performed proximity ligation assays (PLA)^51^. PLA is a technology that extends the capabilities of traditional immunoassays to include direct detection of proteins, protein interactions and modifications with high specificity and sensitivity, allowing detection of interaction distances of as little as ~30 nm (refs 51, 52). A strong and specific PLA signal was detected within the metaphase plate (Fig. 5d). Importantly, the PLA signal is lost by omitting the SMO-1 antibody (Fig. 5e). The PLA signal spreads throughout the metaphase plate as can be appreciated by rotating the image by 90 degrees, towards an anterior-to-posterior view of the metaphase plate (Fig. 5f). Interestingly, the PLA signal is lost during anaphase and is recovered in late anaphase/telophase within the spindle midzone (Fig. 5g). These results show that SUMO and AIR-2 reside in close proximity specifically on chromosomes during metaphase and on the central spindle during anaphase.

**Figure 6:**
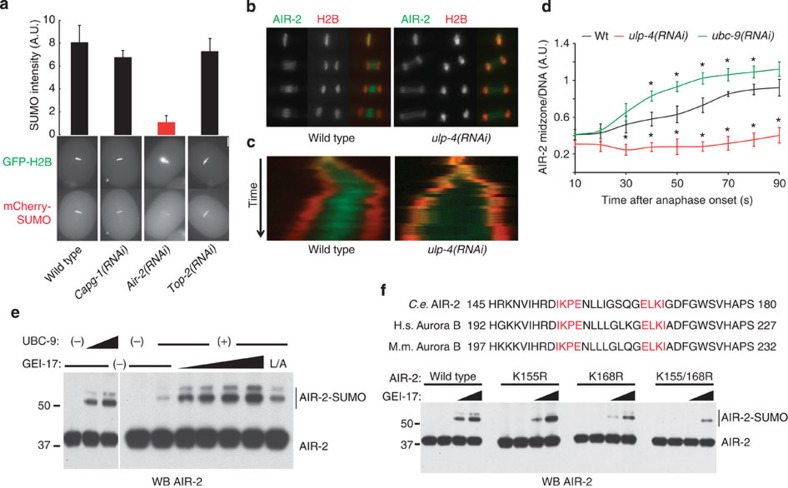
Sumoylation affects AIR-2^Aurora B^ localization during anaphase. (**a**) mCherry-SUMO(GG) and GFP-tagged H2B expressing embryos were fed with bacteria containing the indicated RNAi. Only *air-2* depletion causes a significant decrease in the SUMO signal (red column). Data represent mean and s.d. from three embryos. Scale bar, 10 μm. (**b**) ULP-4 is required for AIR-2 translocation from chromatin to the spindle midzone. Embryos expressing mCherry-H2B and GFP-AIR-2 were analysed. (**c**) Kymographs prepared from the same movies as the images in (**b**). (**d**) UBC-9 and ULP-4 exert opposite effects on AIR-2 localization to the midzone. Localization was quantified as the AIR-2 fluorescence intensity in the midzone relative to the intensity on DNA. *P<0.05 (two-tailed Student’s *t*-test, *n*=5). (**e**) AIR-2 *in vitro* sumoylation reactions were performed. For the GEI-17 dose-response, UBC-9 was used at 200 nM and GEI-17 at 50, 100 and 250 nM. (**f**) Sequence alignment of *C. elegans* AIR-2 and its human and mouse orthologs, Aurora B, bearing the two putative SUMO modification sites (highlighted in red). *In vitro* sumoylation reactions were performed as in (**e**), with limiting amounts of UBC-9 and using 100 and 250 nM GEI-17 using wild-type AIR-2 and mutants.

The topoisomerase II orthologue, TOP-2 has previously been shown to be SUMO modified in mitosis^21^, whereas the Condensin I subunit CAPG-1 exhibits the same localization pattern as SUMO and AIR-2 in mitosis^47^,^48^. Neither *top-2* nor *capg-1* depletion altered the intensity of SUMO at metaphase (Fig. 6a) or the dynamic behaviour of SUMO throughout mitosis (Supplementary Movies 9 and 10). However, in the absence of AIR-2, the SUMO signal associated with metaphase chromosomes was diminished by 80% (Fig. 6a, red column, Supplementary Movie 11). This was also true for endogenous SUMO, as determined by immunostaining (Supplementary Fig. 7). Depletion of *ulp-4* prevented AIR-2 from localizing to the spindle midzone (Fig. 6b,c, Supplementary Movies 12 and 13). While *ulp-4* depletion impaired the localization of AIR-2 in the spindle, depletion of *ubc-9* increased AIR-2 levels in the spindle midzone (Fig. 6d). Detailed analysis of AIR-2 localization throughout mitosis in *ulp-4(RNAi)* embryos showed that, although AIR-2 fails to localize to the spindle midzone during anaphase, it accumulates in the midbody during telophase, which would explain the lack of apparent cytokinesis defect (Supplementary Fig. 8).

### AIR-2 is SUMO-modified *in vitro* in a GEI-17-dependent manner

*In vitro* conjugation reactions showed that AIR-2, like its orthologue Aurora B, is modified by SUMO and the modification is stimulated by GEI-17 in a dose–response manner (Fig. 6e). Moreover, the L/A mutation of the SP-RING within GEI-17 drastically diminishes its SUMO E3 activity. The AIR-2 orthologue in mammals, Aurora B, was previously shown to be modified by SUMO at Lys 207 in mice and Lys 202 in humans^53^,^54^. As shown in Fig. 6f, not only did we detect SUMO conjugation at Lys 155 (equivalent to Lys 202/207 in Aurora B^53^,^54^), but also at Lys 168 (equivalent to Lys 215/220 in Aurora B). Mutation of the two sites decreases the GEI-17-stimulated conjugation by ≥75% (Fig. 6e, ‘K155/168R’ versus ‘wild type’). This putative new SUMO conjugation site resides within an inverted SUMO consensus motif^55^.

## Discussion

**Figure 7:**
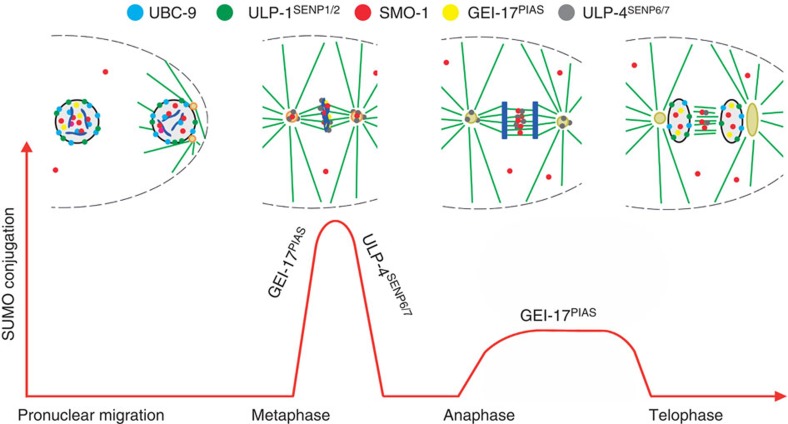
Model. Schematic summarizing the localization of the studied sumoylation pathway components highlighting the dynamic behaviour of SUMO conjugation and the contribution of the SUMO E3 ligase GEI-17^PIAS^ and the SUMO protease ULP-4^SENP6/7^.

We have shown that during the first embryonic cell cycle in *C. elegans*, SUMO is predominantly nuclear at the pronuclear migration stage. Following NEBD and proceeding to metaphase, SUMO is rapidly conjugated to substrates including AIR-2 in an UBC-9- and GEI-17-dependent manner. SUMO is rapidly removed by ULP-4, coincident with AIR-2 localization to the spindle midzone. The summary of the localization of SUMO as well as the enzymes of the sumoylation pathway is depicted in Fig. 7. The dynamic changes in SUMO conjugation are important for chromosome alignment, segregation and, ultimately, for a proper cell cycle progression. Given the previous observation that retention of Aurora B on chromatin leads to a delay in mitotic exit^16^, the mitotic exit delay observed in *ulp-4* depleted embryos could be explained by AIR-2 desumoylation leading to chromatin accumulation. Whether the chromosome alignment and segregation phenotypes are related mechanistically to each other remains to be determined. Also, the effect of SUMO on chromatin condensation and how this affects chromosome dynamics during mitosis will be crucial issues to address in the future. It is worth noting that, in human cells, SUMO proteases have been shown to regulate chromosome behaviour in mitosis^30^,^33^.

In spite of being unable to directly detect AIR-2 sumoylation in worms, the available evidence suggests that AIR-2 is indeed modified by SUMO. First, AIR-2 and SUMO co-localize and SUMO localization depends on AIR-2 (Fig. 5a). Second, AIR-2 is modified *in vitro* and, like mammalian Aurora B^53^, this modification is stimulated by the PIAS-like SUMO E3 GEI-17 (Fig. 6e,f). Third, GEI-17 localizes to the metaphase plate, the exact same place of the AIR-2/SUMO co-localization. Fourth, the use of the PLA in *C. elegans* embryos demonstrates that AIR-2 and SUMO are within ~30/40 nm of each other^51^,^52^. Most strikingly, during anaphase some degree of SUMO/AIR-2 co-localization is observed through conventional immunostaining (Fig. 5a), but no PLA signal is detected (Fig. 5g). Considering the difficulties associated with detecting SUMO conjugation occurring in a specific localization and for time intervals as small as seconds, the PLA assay is an extremely powerful tool that provides visual proof that two proteins are in very close proximity. Given the additional evidence mentioned previously, the most likely explanation for this close proximity is that AIR-2 is conjugated to SUMO, although alternative explanations would be consistent with these data. PLA assays are likely to be a useful tool in *C. elegans*, a system not always amenable to biochemical characterization of protein interactions and modifications.

The precise mechanism by which Aurora B/AIR-2 is extracted from chromatin during anaphase has been a matter of controversy. Culin-3 has been shown to be necessary for ubiquitylation of Aurora B and to regulate translocation of the chromosomal passenger complexfrom chromosomes to the spindle midzone in anaphase^14^,^17^. In addition, p97/Cdc48 binds to ubiquytilated Aurora B and extracts it from chromatin, allowing chromatin decondensation and nuclear envelope formation^16^. In nematodes, CDC-48.3 binds directly to AIR-2 and inhibits its kinase activity from metaphase through telophase^56^. While *cdc-48.3* was identified as a suppressor of embryonic lethality of a temperature-sensitive allele of *air-2* (ref. 56), we have found that *gei-17* RNAi rescues the embryonic lethality of *air-2* temperature-sensitive mutant at the restrictive temperature (data not shown). As this mutant AIR-2 remains bound to chromatin during anaphase, and considering our data that, both *ubc-9* and *gei-17* depletion increase AIR-2 accumulation in the spindle, it is plausible that *gei-17* depletion allows for AIR-2 to localize to the spindle. The fact that SUMO plays a role in AIR-2 chromatin extraction is indeed interesting in light of previous results showing that the Cdc48 co-factor, Ufd1, bears not only ubiquitin-binding domains but also SIMs^57^,^58^. Although SUMO conjugation is not necessary for AIR-2 localization to chromatin (data not shown), it is plausible that SUMO-modified AIR-2 recruits other downstream proteins involved in chromosome condensation and segregation. Sumoylation might force AIR-2 to be retained in the chromatin during anaphase by stabilizing its interaction with chromatin-bound proteins and/or by inhibiting its spindle localization. The fact that the SUMO protease ULP-4 is found in the spindle midzone might suggest that SUMO modification of AIR-2 (or another protein) releases AIR-2 from the spindle allowing it to bind to chromatin. Recently, C. *elegans* dosage compensation complex components were shown to be SUMO substrates, and a SUMO-SIM network was suggested to play a role in the complex assembly^59^. SUMO conjugation to AIR-2 could play a role in recruiting/stabilizing other proteins associated with chromatin such as the condensin I component, CAPG-1, known to require AIR-2 for its localization at metaphase chromatin^47^. SUMO may play separate roles in the regulation of chromosome congression/alignment, regulation of chromosome segregation and regulation of spindle dynamics. These processes could be linked and share common substrates allowing SUMO conjugation and deconjugation to fine-tune protein function and localization in a spatially and timely regulated manner.

In human cells, the microtubule motor protein CENP-E is modified by SUMO-2/3 and binds to SUMO-2/3 chains, and this is essential for kinetochore localization^33^. *C. elegans* lacks an apparent CENP-E orthologue, but the CENP-F-like proteins HCP-1 and HCP-2 recruit the conserved kinetochore- and microtubule-associated proteins *clasp-1* and *clasp-2* to kinetochores^60^. However, perturbation of SUMO conjugation in *C elegans* does not significantly alter the recruitment of HCP-1 to kinetochores. While Zhang *et al.*^33^ reported that SUMO-1 and SUMO-2/3 modification of different proteins regulate distinct processes, *C. elegans* possesses one SUMO protein, SMO-1, that closely resembles mammalian SUMO-1. In fact, unlike SUMO2/3, SMO-1 is unable to form unanchored chains *in vitro* (data not shown). In contrast, the SUMO protease SENP6 regulates the CENP-H/I/K complex in human cells^30^. Again, components of the CENP-H/I/K appear to be lost in *C. elegans* during evolution, raising the question as to how ULP-4 (the nematode ortholog of SENP6/7) affects mitotic progression. Interestingly, we have shown that ULP-4 localizes to metaphase chromatin and to the spindle midzone, so future efforts will focus on these regions and putative substrate proteins. Our findings suggest that in *C. elegans* sumoylation does not drastically affect kinetochore assembly but rather chromosome condensation. We favour a model in which dynamic sumoylation is essential for proper cell cycle progression through the fine-tuning of different processes, without being absolutely required for the embryo to progress through the first mitotic divisions. The precise role of SUMO conjugation and deconjugation and a detailed scrutiny of the substrates remain to be fully understood.

Importantly, most of the SUMO pathway worm mutants or RNAi treatments lead to severe defects, namely embryonic arrest^3^,^4^,^5^,^6^. The defects caused by altering the fine balance between SUMO conjugation and deconjugation does not lead to mitotic arrest during the first embryonic mitotic division. As spindle checkpoint in the embryo is relatively weak^61^, we favour the hypothesis that the consequence of repeated cycles of chromosome segregation defects would ultimately result in an irreparable DNA damage. This work, together with previous data^22^,^25^,^30^,^33^, stress the importance of a tightly regulated SUMO conjugation and deconjugation balance during mitosis.

## Methods

### Worms

*C. elegans* were maintained according to standard procedures^62^. All transgenic worms were generated by particle bombardment^63^. To generate the GFP-tagged fusion protein, the respective full-length cDNAs were amplified from N2 worms and cloned into PIE-1 regulatory element in a pIC26 vector^45^. Worms expressing mCherry-Histone were derived from OD56 (ref. 64).

### Strains

Strain genotypes are listed in Supplementary Table 3. *Smo-1* genomic DNA was amplified with a reverse primer engineered to delete the last codon (F), as to express a ‘processed’ form of SMO-1 ending in GG. The last codon was mutated to alanine to generate SMO-1(GA). Both sequences were cloned in the Spe I sites of pIC26 for GFP or pAA64 for mCherry. The resulting clones were sequenced and integrated into DP38 [*unc-119 (ed3)*] worms by ballistic bombardment with a PDS-1000/He Biolistic Particle Delivery System (Bio-Rad)^63^. Two colour strains were generated by mating. Males were generated by incubation of L4 worms expressing one fluorescent protein at 31 °C for 8 h and subsequently crossed with hermaphrodite worms expressing the second marker. Double homozygotes were screened under a fluorescent microscope^65^. For a complete list of strains used in this study see Supplementary Table 3.

### RNAi

Bacterial (HT115) clones expressing dsRNA for feeding strains were obtained from a commercial library^66^. Bacteria were grown at 37 °C to OD_600_=0.8, shifted to 20 °C, supplemented with 1 mM IPTG and further incubated for 2 h. Then, they were spread on 6-cm nematode growth media plates supplemented with 1 mM IPTG and incubated for 12 h at 20 °C. L4 worms were then added to plates and fed for 24–32 h before analysis. See Supplementary Table 1.

### Generation of antibodies

Monoclonal antibodies against SMO-1 were generated by Dundee Cell Products. SMO-1 was conjugated *in vitro* to mIRF2 and the mixture was used to immunize mice. After selection of SMO-1 reactive sera by ELISA and dot blot, five different lines were isolated and characterized. High titre tissue culture supernatants were obtained with the CELLine CL 1000 Bioreactor (Sartorius). All clones were tested for specificity and the antibody does not recognize neither IRF2 nor UBC9.

GEI-17, ULP-4(catalytic domain) and full-length AIR-2 were used to immunize rabbits (Moravian Biotech). Best responding sera were used for affinity purification using NHS beads coupled to the antigenic peptide/protein after adsorbing the sera with HT1115 bacterial lysate coupled NHS beads. AIR-2 peptide antibodies were produced by Moravian Biotech using a previously described peptide (CQKIEKEASLRNH)^50^. UBC-9 and SMO-1-modified mIRF2 were used to immunize sheep (Scottish antibody production unit/Scottish National Blood Transfusion Service). For a UBC-9 antibody, the serum was passed through an HT1115 bacterial lysate column and then affinity purification was performed with UBC-9 coupled NHS beads. In the case of sheep anti-SMO-1, murine IRF2 was used as a substrate in an *in vitro* conjugation reaction and the reaction was injected in sheep and serum was first ran through an IRF2 column and then affinity purified using recombinant SMO-1. Affinity-purified peptide antibodies against ULP-1 (1.2) and ULP-4 (4.1) were generated in rabbits (Genescript). For all affinity purifications, pre-immune and post-immune sera were first tested for sensitivity and specificity by dot blot using recombinant proteins.

### Microscopy

Embryos were dissected and mounted in M9 buffer on 2% agarose pads, and images were produced using a widefield DeltaVision Core microscope mounted on a microscope (IX71; Olympus) with a × 60/1.40 Plan Apochromat oil immersion lens (Olympus), a camera (CoolSNAP HQ; Photometrics), and softWoRx software. The exposure time was 0.25 s, and binning was 2 × 2. Movie files were generated as reported^67^. For immunostaining, worms were placed on 4 μl of M9 worm buffer in a poly-D-lysine (Sigma, P1024)-coated slide and a coverslip was gently laid on top. Once the worms extruded the embryos, slides were placed on a metal block on dry ice for >10 min, the coverslip taken off with a scalpel blade, and the samples were fixed in methanol at −20 °C. Embryos were stained using standard procedures with mouse monoclonal antibodies for SMO-1 (clones 6F2/D1 and 8A1/D10), sheep polyclonal antibody for SMO-1, mouse monoclonal antibody for α-tubulin (DM1A; Sigma-Aldrich), rabbit polyclonal Anti-phospho-Histone H3 Ser 10 (Millipore), rabbit polyclonal Anti-ULP-4. See Supplementary Table 2 for antibody concentrations. Secondary antibodies were anti-sheep, anti-mouse or anti-rabbit conjugated to Alexa Fluor 488 or Alexa Fluor 568 (1:1,000 Invitrogen). DNA was visualized with Hoechst 33258 (Life Technologies, 1.5 μg ml^−1^ final concentration in PBS, 0.05% Tween-20). Embryos were mounted in 4% n-propyl-gallate (Sigma), 90% glycerol, in PBS and were imaged using a DeltaVision Core microscope (see above). Each embryo shown is representative of ≥10 embryos observed. For Figs 1c, 2d,e and 3j, Supplementary Figs 3c,d, 5 and 8b, images were acquired using a spinning-disk confocal microscope (MAG Biosystems) mounted on a microscope (IX81; Olympus) with a × 100/1.45 Plan Apochromat oil immersion lens (Olympus), a camera (Cascade II; Photometrics), spinning-disk head (CSU-X1; Yokogawa Electric Corporation) and MetaMorph software (Molecular Devices).

### Quantification of SUMO and AIR-2 signal on DNA

An area of interest was drawn around the DNA (using H2B as a guide). The corresponding GFP image was then used to determine mean values for DNA and cytoplasm. Background intensity was taken in the cytoplasm. For AIR-2 translocation, the midzone/chromatin ratio was determined as follows: ratio=(DNA—background)/(midzone—background). This procedure was applied to all images of the video using ImageJ 1.46r.

### Plasmids

*C. elegans smo-1*, *ubc-9, ulp-4, gei-17 and air-2* cDNAs were cloned in the pHISTEV30a vector that includes an N-terminal hexahistidine tag followed by a TEV protease recognition site. *Smo-1* was mutated to obtain a processed version of the protein [SMO-1(GG)] and cloned using *Nco* I and *Hind* III. The *ulp-4* fragment containing the catalytic domain included nucleotides 433–999 of the coding sequence (NM_063302.4) and was cloned using *Nco* I and *Hind* III. A fragment of the PIAS orthologue *gei-17* (coding for aa 133–509 of isoform f, NP_001021678.3) spanning was inserted in pHISTEV30a using *Nco* I and *Not* I. *Ubc-9* and *air-2* full length cDNAs were cloned in pHISTEV30a using *Nco* I and *Hind* III. The different plasmid DNAs were transformed into BL21 Rosetta bacterial strain and several colonies tested for induction.

### Protein purification

For protein purification, cultures were grown until OD_600_=0.8, cooled down on ice and induced with 0.1 mM IPTG for 16 h at 20 °C. The bacterial cells were harvested by centrifugation (6,200 *g* for 20 min at 4 °C), and the cell pellet was resuspended in 35 ml of lysis buffer (50 mM Tris, 500 mM NaCl, 10 mM imidazole, Complete protease inhibitor cocktail tablet, EDTA-free (Roche), 0.1% Triton X-100, pH 7.5). The bacterial cells were lysed by sonication (Digital Sonifier, Branson) for 4 × 20″ pulses at 50% amplitude, with a 20-second cooling period between pulses. Sample was centrifuged (27,200 g for 45 min at 4 °C) to remove any insoluble material. The supernatant was filtered through a 0.45 μm filter and loaded onto Ni-NTA agarose beads (Qiagen) pre-equilibrated with lysis buffer without Triton X-100 buffer. The column was washed successively with binding buffer (~8 column volumes) and washing buffer (lysis buffer with 30 mM imidazole final concentration, ~8 column volumes), and the fusion protein was then eluted with elution buffer (50 mM Tris, 150 mM NaCl, 200 mM imidazole, 0.5 mM TCEP, pH 7.5). TEV protease was added (1 mg of TEV protease per 100 mg of the fusion protein). The sample was dialysed overnight at 4 °C against 50 mM Tris, 150 mM NaCl, 0.5 mM TCEP, pH 7.5. After ~16 h at 4 °C, imidazole was added to the final concentration of 10 mM (30 mM for GEI-17) and the samples were centrifuged (3,900 g for 15 min at 4 °C) to remove any precipitated material. Supernatant was then passed through the Ni-NTA agarose column pre-equilibrated with 50 mM Tris, 150 mM NaCl, 10 mM imidazole, 0.5 mM TCEP, pH 7.5. The flow-through fraction was collected. This step removed free His_6_-tag, any uncleaved His_6_-tagged protein and the TEV protease (as it is also His_6_-tagged). The flow-through fraction was dialysed overnight at 4 °C against 50 mM Tris, 150 mM NaCl, 0.5 mM TCEP, pH 7.5. The sample was then concentrated using a centrifugal concentrator (Sartorius) with a molecular weight cutoff of 5,000. UBC-9 was further purified using cation exchange (monoS), SMO-1, ULP-4 and GEI-17 using anion exchange (monoQ), and AIR-2 using size exclusion chromatography (Superdex 75). Purified proteins were aliquoted, flash-frozen in liquid nitrogen and stored at −80 °C.

### *In vitro* sumoylation

All reactions were buffered in 50 mM tris-HCl (pH 7.5). SUMO proform processing assays contained 150 mM NaCl, 0.5 mM TCEP, 50 μM SUMO and 100 nM SENP1 or ULP-4 recombinant catalytic domains, and reactions were incubated at 30 °C for 60 min. Conjugation assays contained 5 mM dithiothreitol, 5 mM MgCl_2_, 2 mM ATP, 100 ng of SAE1/SAE2, 1 and 2 μM UBC-9 (reduced to 200 nM for GEI-17-dependent AIR-2 conjugation), ~1 μg of substrate protein, and 5 μg of SUMO and were incubated at 37 °C for 4 h. Chain editing assays were performed by adding 0.5, 1 and 4.5 μM of the catalytic domain of ULP-4 (aa 145–333 in NP_495703.2) for the indicated times, whereas SMO-1 processing was performed for 2 h at 37 °C using 1 μM ULP-4 CD. For the chain editing assays, SMO-1 chains were formed in the presence of GEI-17 and purified by size exclusion chromatography (Superdex 200).

### Duolink *in situ* PLA

PLAs were performed using primary antibodies directly coupled to the PLA probes or using secondary antibody PLA probes (Sigma-Aldrich). For the direct PLA, ~35 worms were placed on a drop of 4 μl of M9 worm buffer in a poly-D-lysine-coated slide and a coverslip was gently laid on top. Once the worms extruded the embryos, slides were freeze-cracked: placed on a metal block on dry ice for >10 min, the coverslip taken off with a scalpel blade, and the samples were fixed in methanol at −20 °C for 30 min. After sequential washes (5 min each) with PBS+0.5% Triton X-100, PBS+0.1% Tween-20 and PBS, slides were incubated with the monoclonal α-SMO-1 (6F2/D1, 10 μg ml^−1^) and α-AIR-2 (10 μg ml^−1^), both previously coupled to the PLA oligonucleotide arms using the Duolink *in situ* Probemaker overnight at 4 °C. Ligation and amplification were performed as detailed by the manufacturer. Controls omitting either of the antibodies gave no PLA signal. For indirect PLA, the same primary antibodies were used (unlabelled) and after an overnight incubation at 4 °C slides were incubated with anti-mouse and anti-rabbit secondary antibodies coupled to the PLA oligonucleotide probes. Ligation and amplification were performed as detailed by the manufacturer. In both cases, slides were incubated in Hoechst 33258 at 1.5 μg ml^−1^ in PBS+0.1% Tween-20 for 5 min. Slides were mounted in 4% *n*-propyl-gallate, 90% glycerol, in PBS and were imaged using a DeltaVision Elite microscope.

## Author contributions

F.P. conceived the project; performed the cloning and generated all strains, antibodies and recombinant proteins; designed, performed and interpreted experiments; and wrote the manuscript. R.S. designed, performed and interpreted experiments and assisted with microscopy techniques. E.P. assisted with general worm techniques and performed initial bombardments. A.A. assisted with general worm techniques and carried out initial immunostaining experiments. J.J.B. contributed tools and reagents. A.G. supervised the project and provided advise on general *C. elegans* work. R.T.H. designed and interpreted experiments, supervised the project and wrote the manuscript. All authors commented on the manuscript.

## Additional information

**How to cite this article**: Pelisch, F. *et al.* Dynamic SUMO modification regulates mitotic chromosome assembly and cell cycle progression in *Caenorhabditis elegans*. *Nat. Commun.* 5:5485 doi: 10.1038/ncomms6485 (2014).

## Supplementary Material

Supplementary Information: Supplementary Figures, Supplementary Tables. Supplementary Figures 1-8, Supplementary Tables 1-3.

Supplementary Movie 1. GFP-H2B/GFP- γ-tubulin expressing embryos were recorded. Left panel, wild type embryo; Right panel, *smo-1(RNAi)*. Images were acquired every 10 s using a DeltaVision microscope.

Supplementary Movie 2. mCherry-SMO-1(GG)/GFP-H2B expressing embryos were recorded. Left panel, mCherry; Right panel, GFP. Images were acquired every 10 s using a DeltaVision microscope.

Supplementary Movie 3. mCherry-SMO-1(GG)/GFP-H2B expressing *ubc-9(RNAi)* embryos were recorded using a Delta Vision microscope. Left panel, mCherry; Right panel, GFP. Images were acquired every 10 s using a DeltaVision microscope.

Supplementary Movie 4. *mms-21(RNAi)* embryos expressing mCherry-SMO-1(GG)/GFP-β-tubulin were recorded. Left panel, mCherry; middle panel, GFP; Right panel, merge. Images were acquired every 10 s.

Supplementary Movie 5. *mms-21(RNAi)* embryos expressing mCherry-SMO-1(GG)/GFP-β-tubulin were recorded. Left panel, mCherry; middle panel, GFP; Right panel, merge. Images were acquired every 10 s.

Supplementary Movie 6. mCherry-SMO-1(GG)/GFP-H2B expressing embryos were recorded. Left panel, mCherry; middle panel, GFP; Right panel, merge. Images were acquired every 10 s.

Supplementary Movie 7. mCherry-SMO-1(GG)/GFP-H2B-expressing *ulp-4(RNAi)* embryos were recorded. Left panel, mCherry; middle panel, GFP; Right panel, merge. Images were acquired every 10 s.

Supplementary Movie 8. mCherry-SMO-1(GG)/GFP-AIR-2 expressing embryos were recorded. Left panel, mCherry; right panel, GFP. Images were acquired every 10 s.

Supplementary Movie 9. mCherry-H2B/GFP-AIR-2 expressing embryos were recorded. Left panel, mCherry; right panel, GFP. Images were acquired every 10 s. Movie 9, *top-2(RNAi)*; Movie 10, *capg-1(RNAi)*; Movie 11, *air-2(RNAi)*.

Supplementary Movie 10. mCherry-H2B/GFP-AIR-2 expressing embryos were recorded. Left panel, mCherry; right panel, GFP. Images were acquired every 10 s. Movie 9, *top-2(RNAi)*; Movie 10, *capg-1(RNAi)*; Movie 11, *air-2(RNAi)*.

Supplementary Movie 11. mCherry-H2B/GFP-AIR-2 expressing embryos were recorded. Left panel, mCherry; right panel, GFP. Images were acquired every 10 s. Movie 9, *top-2(RNAi)*; Movie 10, *capg-1(RNAi)*; Movie 11, *air-2(RNAi)*.

Supplementary Movies 12. mCherry-H2B/GFP-AIR-2 expressing embryos were recorded. Left panel, GFP; middle panel, mCherry; right panel, merge. Images were acquired every 10 s. Movie 12, *wild type*; Movie 13, *ulp-4(RNAi)*.

Supplementary Movies 13. mCherry-H2B/GFP-AIR-2 expressing embryos were recorded. Left panel, GFP; middle panel, mCherry; right panel, merge. Images were acquired every 10 s. Movie 12, *wild type*; Movie 13, *ulp-4(RNAi)*.
