# Author Correction: Dynamic SUMO modification regulates mitotic chromosome assembly and cell cycle progression in *Caenorhabditis elegans*

**DOI:** 10.1038/s41467-022-35079-7

**Published:** 2022-11-24

**Authors:** Federico Pelisch, Remi Sonneville, Ehsan Pourkarimi, Ana Agostinho, J. Julian Blow, Anton Gartner, Ronald T. Hay

**Affiliations:** 1 Centre for Gene Regulation and Expression, College of Life Sciences, University of Dundee, Dundee, DD1 5EH UK; 2 Present Address: MRC Protein Phosphorylation and ubiquitylation Unit, University of Dundee, Dundee, DD1 5EH UK; 3 Present Address: Memorial Sloan Kettering Cancer Center, New York, NY 10065 USA; 4 Present Address: Department of Cell and Molecular Biology, Karolinska Institutet, Stockholm, S-171 77 Sweden

Correction to: *Nature Communications* 10.1038/ncomms6485, published online 05 December 2014

The original version of this article contained an error in Fig. 6, in which the leftmost panel in Fig. 6f was also mistakenly inserted into the leftmost panel of Fig. 6e. The correct version of Fig. 6 is:
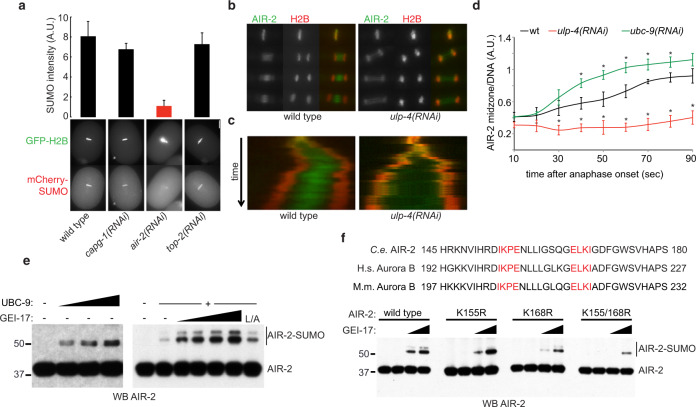


As a consequence of the error in Fig. 6, the legend of Fig. 6e, f incorrectly reads ‘(**e**) AIR-2 in vitro sumoylation reactions were performed. For the GEI-17 dose response, UBC-9 was used at 200 nM and GEI-17 at 50, 100 and 250 nM. (**f**) Sequence alignment of *C. elegans* AIR-2 and its human and mouse orthologs, Aurora B, bearing the two putative SUMO modification sites (highlighted in red). In vitro sumoylation reactions were performed as in (**e**), with limiting amounts of UBC-9 and using 100 and 250 nM GEI-17 using wild-type AIR-2 and mutants.’

This should read ‘(**e**)’ AIR-2 in vitro sumoylation reactions were performed. On the left panel, UBC-9 was used at 200, 400, and 1000 nM. For the GEI-17 dose response (right blot), UBC-9 was used at 200 nM and GEI-17 at 50, 100, 250 and 500 nM. (**f**) Sequence alignment of *C. elegans* AIR-2 and its human and mouse orthologs, Aurora B, bearing the two putative SUMO modification sites (highlighted in red). In vitro sumoylation reactions were performed as in (**e**), with limiting amounts of UBC-9 (100 nM) and using 100 and 250 nM GEI-17 using wild-type AIR-2 and mutants.’, where ‘GEI-17 at 50, 100, 250 and 500 nM’ replaces ‘GEI-17 at 50, 100 and 250 nM’ and ‘limiting amounts of UBC-9 (100 nM)’ replaces ‘limiting amounts of UBC-9’.

The errors have not been corrected in the PDF or HTML versions of the article.

