# Supplementary material for: Dynamic SUMO modification regulates mitotic chromosome assembly and cell cycle progression in Caenorhabditis elegans
Source: Nat Commun. 2014 Dec 5;5:5485. doi: 10.1038/ncomms6485 (PMC4268692; doi:10.1038/ncomms6485)
Supplement: Supplementary Information: Supplementary Figures, Supplementary Tables. — Supplementary Figures 1-8, Supplementary Tables 1-3. [file ncomms6485-s1.pdf]

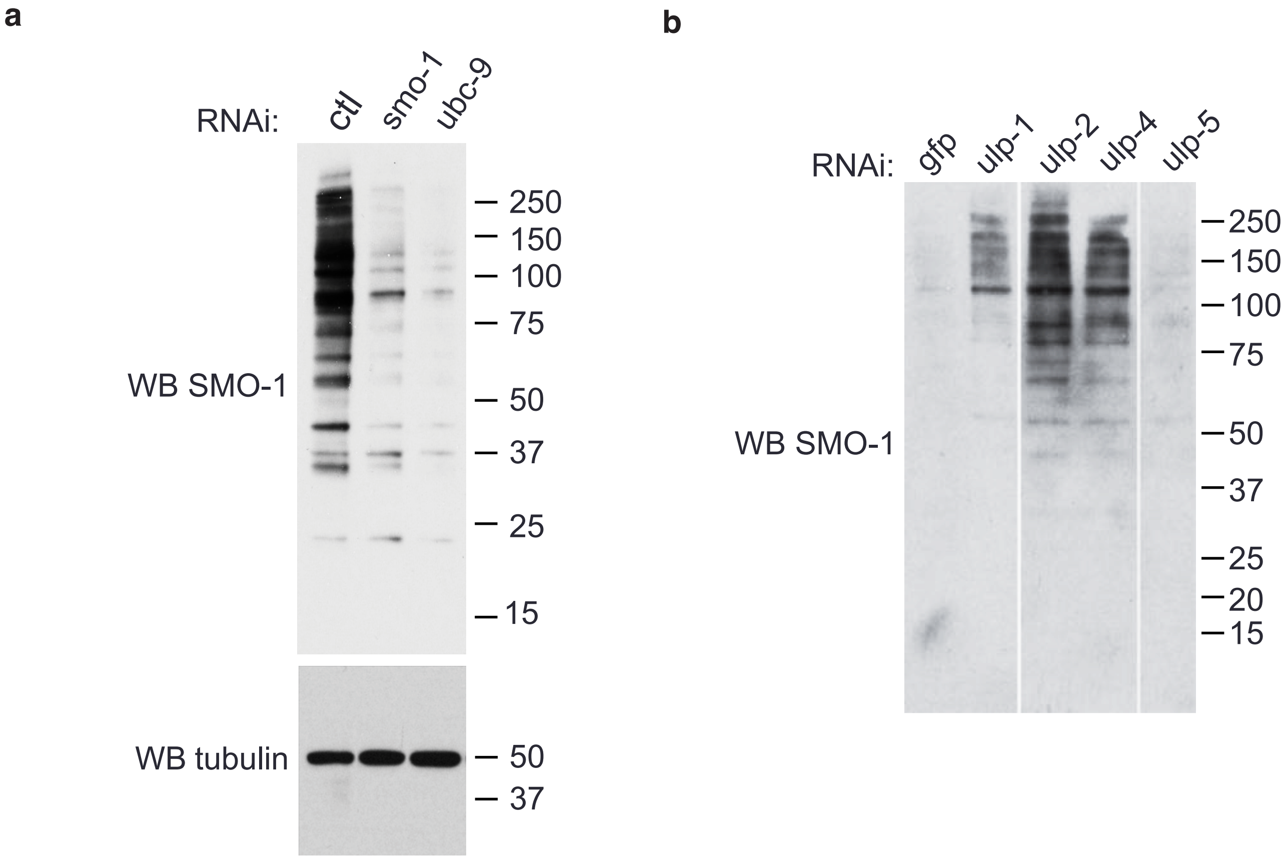

Supplementary Figure 1. N2 worms at the L4 stage were plated on the indicated RNAi plates and incubated for 48 h at 25°C. Embryos were prepared by bleaching and resuspended in SDS sample buffer. Samples were sonicated in a water bath (Bioruptor) at high setting for 2x30" with 30" off in between pulses and then boiled for 5 min. Samples were loaded on 4-12% SDS gels and run in MES buffer. Anti-SMO-1 (6F2) and anti-tubulin (DM1) were used for western blotting.

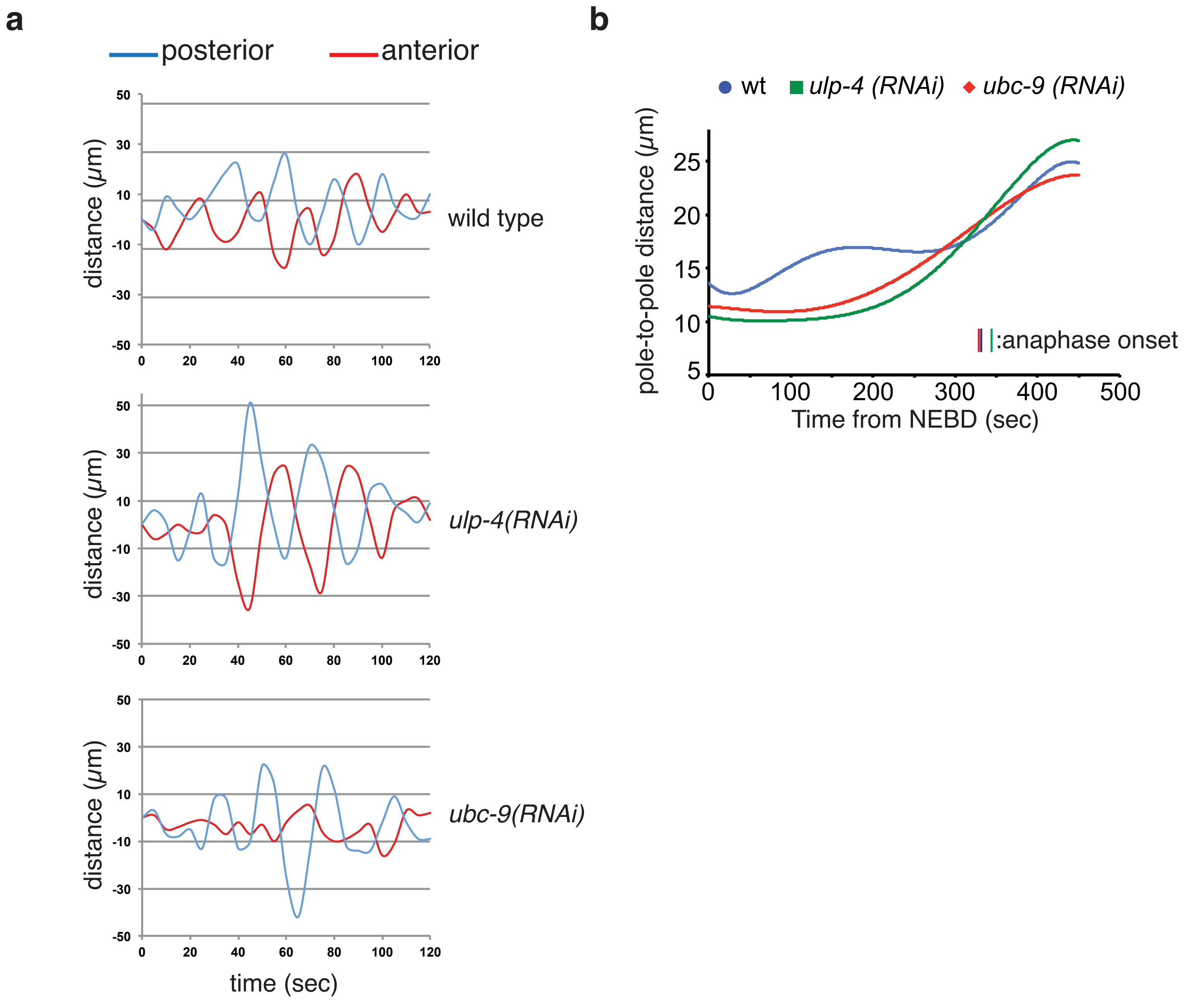

Supplementary Figure 2. (a) Embryos expressing GFP-H2B and GFP-γ-tubulin (Strain TH32) were used to measure spindle oscillations (rocking). Distance from each centrosome to a line drawn along the long axis of the embryo was measured every 5 seconds and the results are plotted. (b) The same strain as in (a) was used to measure the distance between spindle poles.

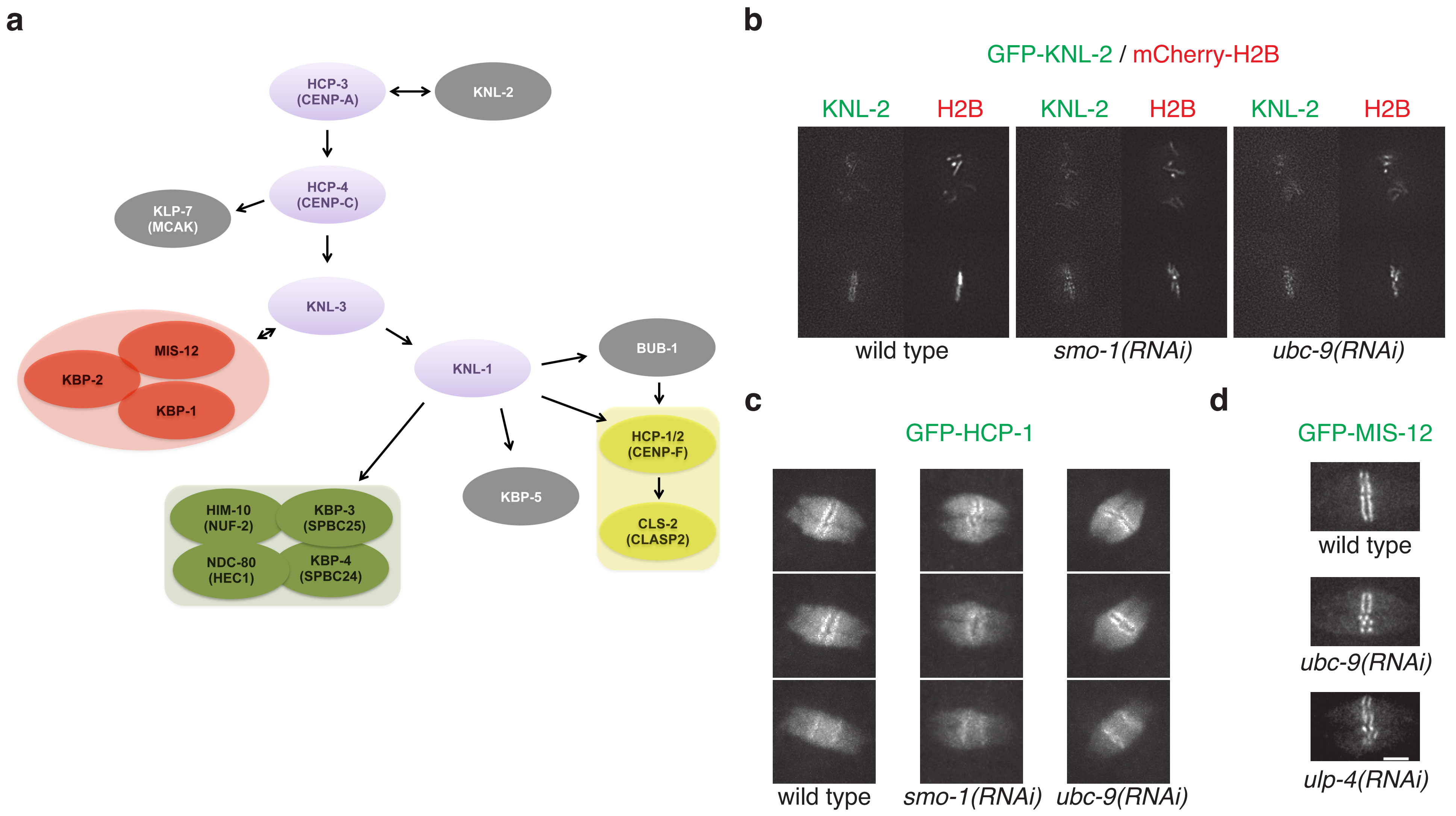

Supplementary Figure 3. Kinetochore proteins are recruited without major abnormalities in the absence of a functional sumoylation pathway. (a) Schematic of the kinetochore protein network in *C. elegans*. (b) Wild type, *smo-1(RNAi)*, and *ubc-9(RNAi)* embryos expressing GFP-KNL-2/mCherry-H2B were analysed for GFP-KNL-2 recruitment to chromatin and localisation pattern. (c) Wild type, *smo-1(RNAi)*, and *ubc-9(RNAi)* embryos expressing GFP-HCP-1 (Strain OD7) were analysed for GFP-HCP-1 localisation pattern on metaphase/anaphase chromosomes. (d) Wild type, *ulp-4(RNAi)*, and *ubc-9(RNAi)* embryos expressing GFP-MIS-12 (Strain OD8) were analysed for GFP-MIS-12 localisation pattern on metaphase/anaphase chromosomes.

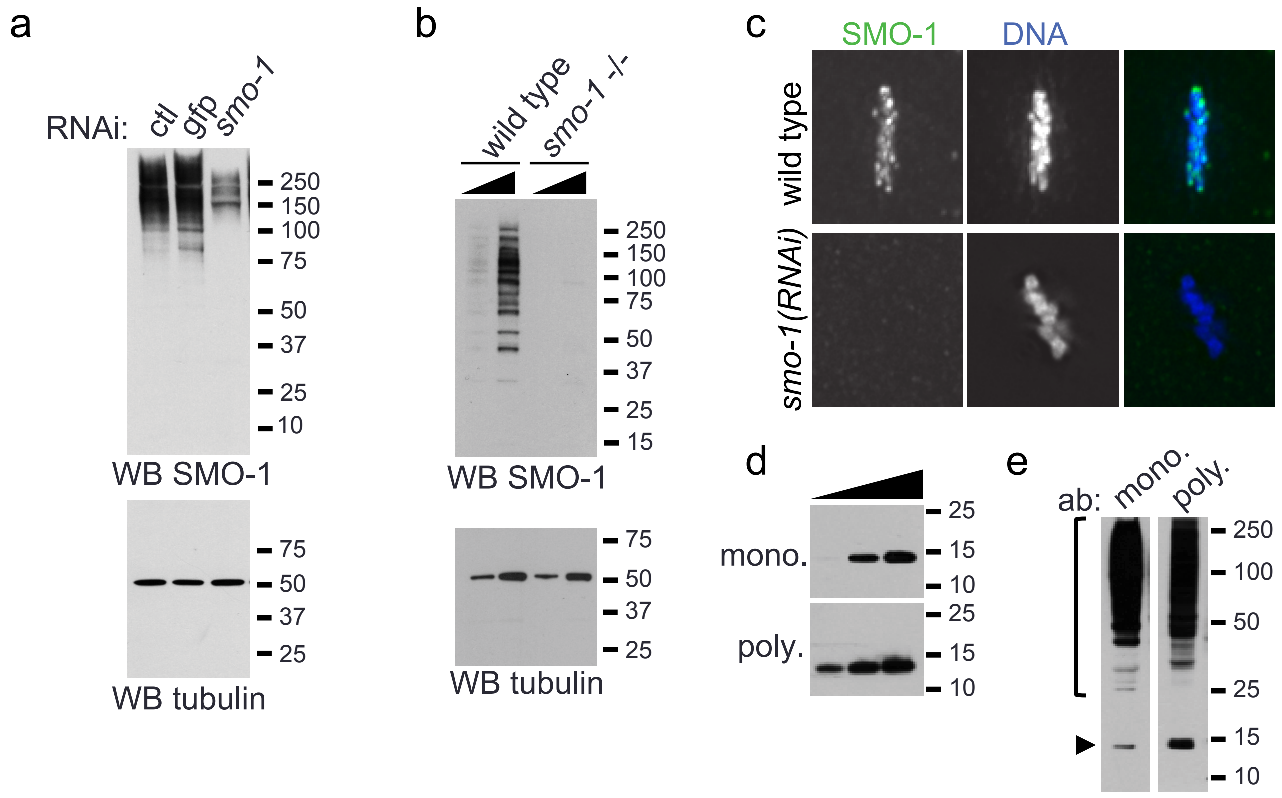

Supplementary Figure 4. Analysis of the SMO-1 antibodies developed. (a) N2 worms at the L4 stage were plated on the indicated RNAi plates and incubated for 48 h at 25°C. Embryos were prepared by bleaching and resuspended in SDS sample buffer. Samples were sonicated in a water bath (Bioruptor) at high setting for 2x30” with 30” off in between pulses and then boiled for 5 min. Samples were loaded on 4-12% SDS gels and run in MES buffer. Anti-SMO-1 (6F2) and anti-tubulin (DM1) were used for western blotting. (b) Same as in (a), but N2 embryo lysate was compared to smo-1 embryo lysate. (c) Immunostaining of embryos was performed as described in the methods section. A mouse monoclonal anti-SMO-1 (6F2) was used and Hoechst 33258 was used to stain DNA. (d) Increasing amounts of recombinant SMO-1 were loaded on a 4-12% SDS gel and western blot was performed with a mouse monoclonal anti-SMO-1 (6F2, top blot) or a sheep polyclonal anti-SMO-1 (bottom blot). (e) Same as in (d) but with N2 worm lysate instead of recombinant SMO-1. Black arrowhead indicates free, unconjugated SMO-1 and the bracket indicates the SMO-1 conjugates.

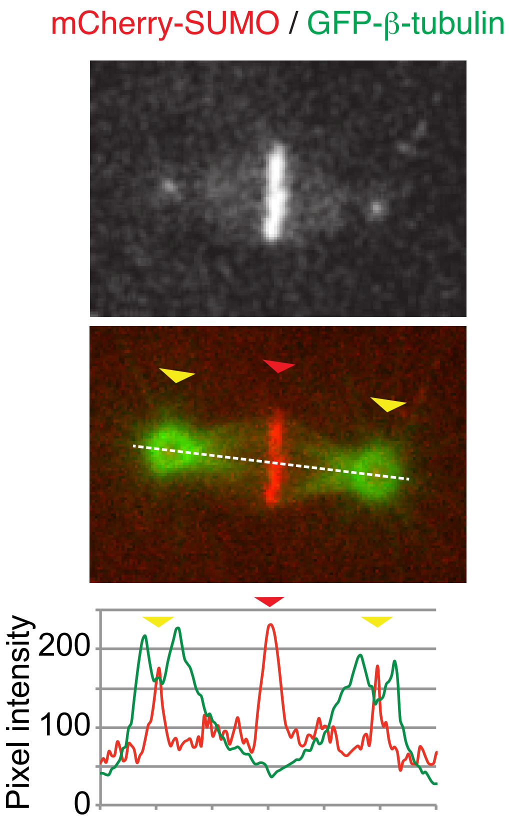

Supplementary Figure 5. SUMO localises to metaphase chromosomes and centrosomes. A metaphase image from a time lapse using the strain expressing mCherry-SUMO together with β-tubulin is shown. The image was acquired with a spinning disk confocal microscope. A line crossing the spindle poles (yellow arrowheads) and the metaphase plate (red arrowhead) was arbitrarily drawn, and pixel intensity values were taken and used for the graph below.

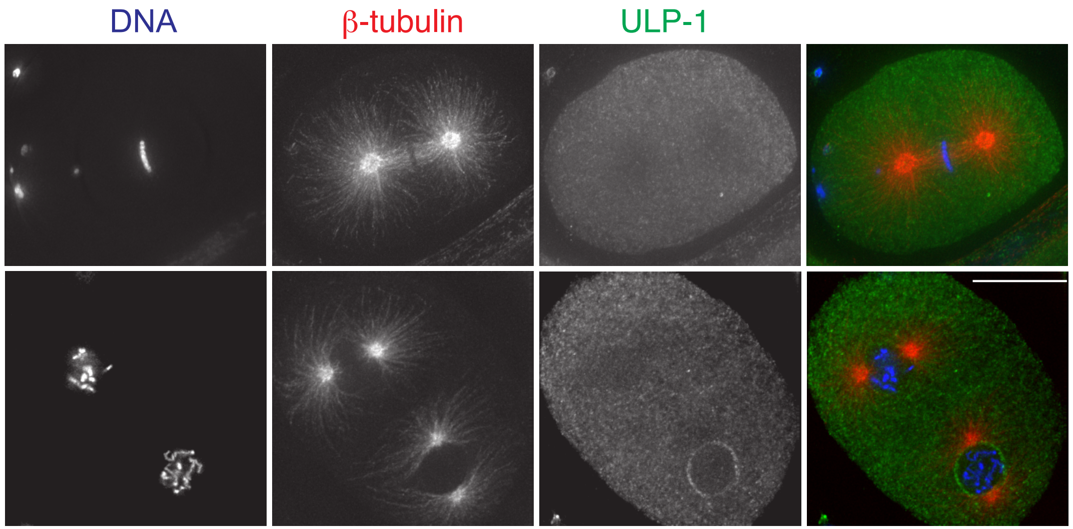

Supplementary Figure 6. Immunostaining of embryos was performed as described in the methods section. A rabbit polyclonal anti-ULP-1 and mouse monoclonal anti-tubulin were used. Hoechst 33258 was used to stain DNA. Top row shows an embryo in metaphase at the 1-cell stage and the middle row shows an embryo with the AB cell in prometaphase (after NEBD) and the P1 cell in prophase (before NEBD). Scale bar, 15 μm.

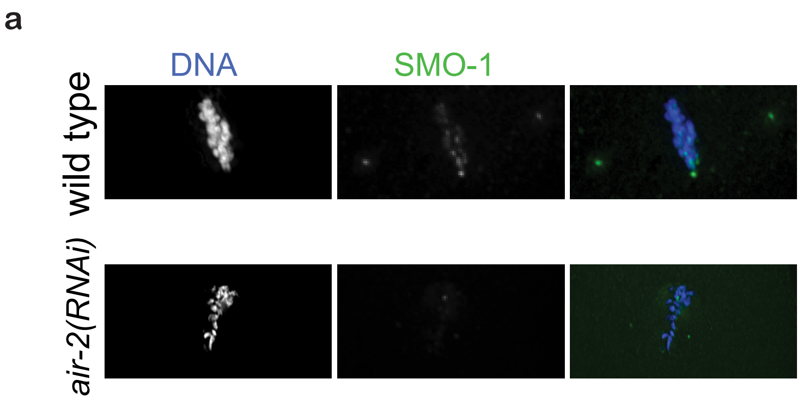

Supplementary Figure 7. Images of the metaphase plate were taken from wild type (top) and *air-2(RNAi)* (bottom) embryos. Immunostaining of embryos was performed as described in the methods section using a mouse monoclonal anti-SMO-1 (6F2). Hoechst 33258 was used to stain DNA.

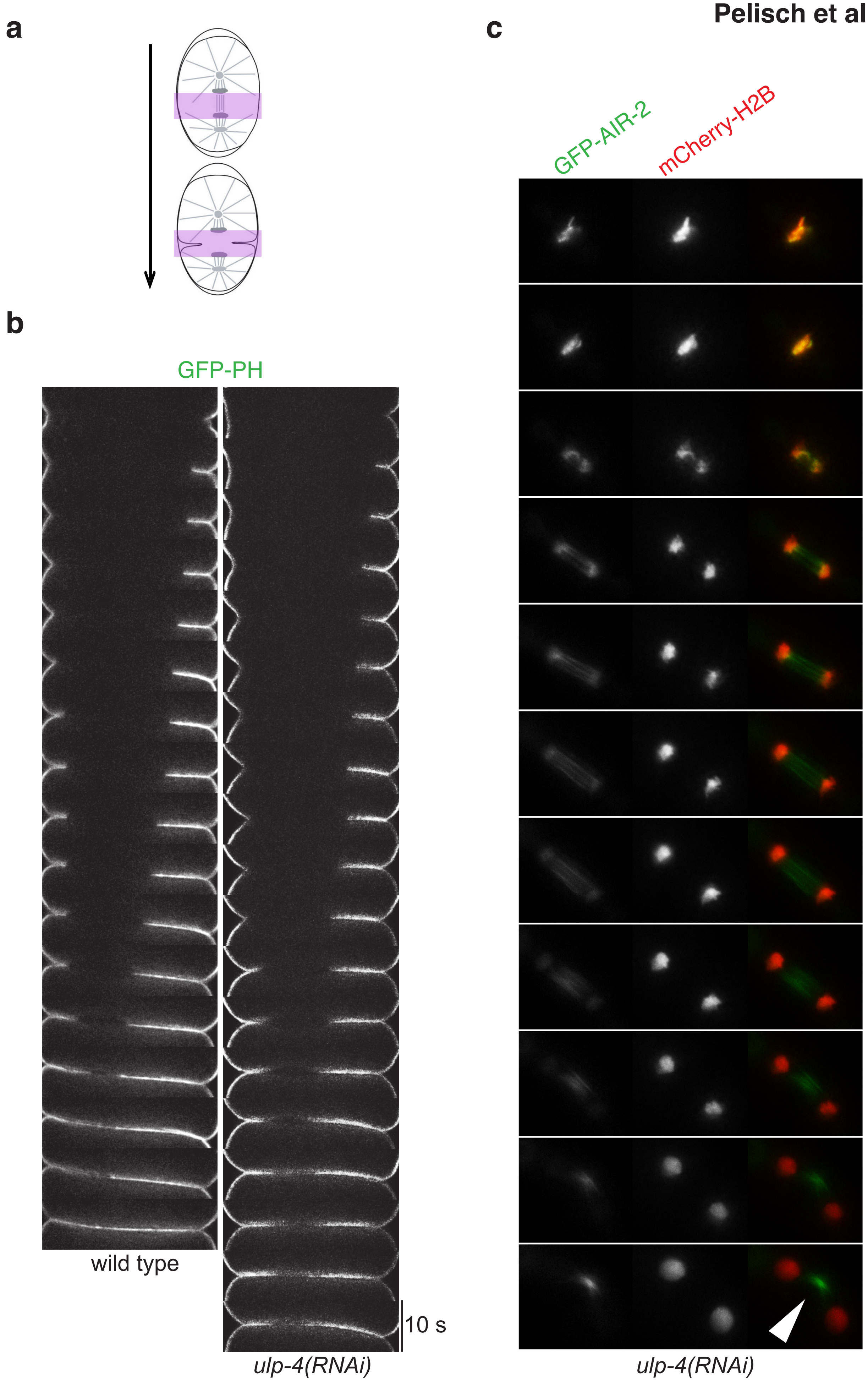

Supplementary Figure 8. (a) Schematic showing and embryo before and after furrow ingression. The area highlighted in pink was selected from time-lapse movies of both wild type and *ulp-4(RNAi)* embryos expressing GFP-PH (as a membrane marker), and stacked together to get the montage shown in **(b)**. **(c)** *ulp-4(RNAi)* embryos expressing GFP-AIR-2 and GFP-H2B were recorded and the montage shows stacks from metaphase to cytokinesis. The white arrowhead points to the GFP signal on the mid-body.

Supplementary Table 1 (RNAi)

RNAi	GenePairs Name	SourceBioscience location
smo-1	K12C11.2	I-1013
ubc-9	F29B9.6	IV-2K06
gei-17	W10D5.3	I-4D09
mms-21	ZK1248.11.1	II-10O15
ulp-1	T10F2.3	III-2N21
ulp-2	Y38A8.3	II-4K17
ulp-4	C41C4.6	II-6A07
ulp-5	K02F2.4	I-3I02
top-2	K12D12.1	II-8E05
capg-1	F29D11.2	I-3D12
air-2	B0207.4	I-9A05

Bacterial clones expressing the corresponding dsRNA were obtained from a commercial library [1].

1. Kamath, R.S. *et al.* Systematic functional analysis of the Caenorhabditis elegans genome using RNAi. *Nature* **421**, 231-237 (2003).

Supplementary Table 2 (antibodies)

Antigen	Species	WB	IF
AIR-2 (Cys-QKIEKEASLRNH)	Rabbit	2 μg/ml	5 μg/ml
ULP-1(1.2) (Cys-ASRRTTPRFTQKNM)	Rabbit	N.D.	0.5 μg/ml
UBC-9 (full length)	Sheep	2 μg/ml	10 μg/ml
GEI-17 (aa 133-509 of isoform f)	Rabbit	N.D.	2 μg/ml
ULP-4(4.1) (Cys-PKDLDNFDFAREYP)	Rabbit	N.D.	10 μg/ml
ULP-4(CD) (aa 145-382)	rabbit	N.D.	5 μg/ml
SMO-1 (full length)	Mouse (6F2)	1 μg/ml	5 μg/ml
SMO-1 (full length)	Sheep	0.5 μg/ml	1 μg/ml
Tubulin (DM1A, Sigma)	Mouse	0.5 μg/ml	2.5 μg/ml

Table 2. List of antibodies used in this study, including working concentrations for western blot (WB) and immunofluorescence (IF). The species in which each antibody was raised as well as the antigen used are indicated. Affinity purification of the antibodies is described in the Methods section.

Supplementary Table 3. Fluorescent strains used in this study.

Fluorescent Protein(s)	Strain	genotype	source
GFP-H2B / GFP-γ-tubulin	TH32	*ddIs6 [tbg-1::GFP + unc-119(+)], ruIs32 [pie-1::GFP::H2B + unc-119(+)] III*	CGC
mCherry-H2B	OD56	*ltIs37 [pAA64; pie-1p::mCherry::his-58 + unc-119(+)]*	CGC
mCherry-H2B / GFP-AIR-2	TG3825	*ltIs37 [pie-1p::mCherry::his-58 (pAA64) + unc-119(+)], ojIs50 [pie-1p::GFP::air-2 + unc-119(+)], unc-119 (ed3)*	Gartner Lab
mCherry-H2B / GFP-KNL-2	TG3826	*tIs37 [pie-1p::mCherry::his-58 (pAA64) + unc-119(+)], ltIs22[pPM3; pie-1::GFP-TEV-STag::KNL-2 + unc-119(+)], unc-119 (ed3)*	Gartner Lab
GFP-KNL-2	OD31	*ltIs22[pPM3; pie-1::GFP-TEV-STag::KNL-2 + unc-119(+)]*	
GFP-H2B	AZ212	*ruIs32 [pie-1::GFP::H2B + unc-119(+)], unc-119 (ed3)*	CGC
GFP-AIR-2	WH371	*ojIs50 [pie-1p::GFP::air-2 + unc-119(+)], unc-119 (ed3)*	CGC
GFP-β-tubulin	AZ244	*ruIs57[pie-1::GFP::tubulin + unc-119(+)], unc-119 (ed3)*	CGC
mCherry-SMO-1(GG)	FGP1	*fgpIs20[pAA64; Ppie-1 mCherry::smo-1(GG) unc-119(+)], unc-119 (ed3)*	this study
mCherry-SMO-1(GA)	FGP2	*fgpIs21[pAA64; Ppie-1 mCherry::smo-1(GA) unc-119(+)], unc-119 (ed3)*	this study
mCherry-SMO-1(FV/AA, GG)	FGP6	*fgpIs22[pAA64; Ppie-1 mCherry::smo-1(FV/AA, GG) unc-119(+)], unc-119 (ed3)*	this study
GFP-SMO-1(GG)	FGP3	*fgpIs23[pIC26; Ppie-1 LAP::smo-1(GG) unc-119(+)], unc-119 (ed3)*	this study
GFP-SMO-1(GA)	FGP4	*fgpIs24[(pIC26); Ppie-1 LAP::smo-1(GA) unc-119(+)], unc-119 (ed3)*	this study
GFP-SMO-1(GG) / mCherry-H2B	FGP9	*fgpIs23[pIC26; pie-1/GFP-TEV-S-Tag::smo-1(GG) unc-119(+)], unc-119 (ed3), ltIs37 [pAA64; pie-1p::mCherry::his-58 + unc-119(+)]*	this study
GFP-SMO-1(GA) / mCherry-H2B	FGP10	*fgpIs24[pIC26; pie-1/GFP-TEV-S-Tag::smo-1(GA) unc-119(+)], unc-119 (ed3), ltIs37 [pAA64; pie-1p::mCherry::his-58 + unc-119(+)]*	this study
GFP-HCP-1	OD7	*ltIs3 [pIC31; pie-1::hcp-1::GFP-TEV-S-Tag + unc-119 (+)]*	CGC
GFP-HCP-1 / mCherry-H2B	FGP11	*ltIs3 [pIC31; pie-1::hcp-1::GFP-TEV-S-Tag + unc-119 (+)], ltIs37 [pAA64; pie-1p::mCherry::his-58 + unc-119(+)]*	This study
GFP-MIS-12	OD8	*ltIs4 [pIC32; pie-1/GFP-TEV-S-Tag::mis-12 + unc-119 (+)]*	CGC
mCherry-SMO-1(GG) / GFP-β-tubulin	FGP7	*ruIs57[pie-1::GFP::tubulin + unc-119(+)], fgpIs20[pAA64; Ppie-1 mCherry::smo-1(GG) unc-119(+)], unc-119 (ed3)*	this study
mCherry-SMO-1(GG) / GFP-AIR-2	FGP5	*ltIs14[pASM05: pie-1::GFP-TEV-STag::air-2 + unc-19(+)], fgpIs20[pAA64; Ppie-1 mCherry::smo-1(GG) unc-119(+)], unc-119 (ed3)*	this study
mCherry-H2B / GFP-PH(PLC1delta1)	OD95	*ltIs37 [pAA64; pie-1::mCherry::HIS-58 + unc-119(+)] IV. ltIs38 [pAA1; pie-1::GFP::PH(PLC1delta1) + unc-119(+)]*	CGC
mCherry-SMO-1(GG) / GFP-H2B	FGP8	*ruIs32 [pie-1::GFP::H2B + unc-119(+)], fgpIs20[pAA64; Ppie-1 mCherry::smo-1(GG) unc-119(+)], unc-119 (ed3)*	this study

Mutations and rearrangements used were as follows:

LG I: *smo-1(ok359)/szT1[lon-2(e678)] I; +/szT1 X*

LG I: *gei-17(tm2723)/hT2[bli-4(e937) let-?(q782) qIs48]*

LG IV: *ubc-9(tm2610) IV/ nT1[qIs51], nT1[unc-?(n754) let-? qIs50]*
